# Supplementary material for: Metabolomics Approach on Non-Targeted Screening of 50 PPCPs in Lettuce and Maize
Source: Molecules. 2022 Jul 23;27(15):4711. doi: 10.3390/molecules27154711 (PMC9330060; doi:10.3390/molecules27154711)
Supplement: Supplementary file 1 [file molecules-27-04711-s001.zip › molecules-1807056-supplementary.pdf]

Supplementary Materials

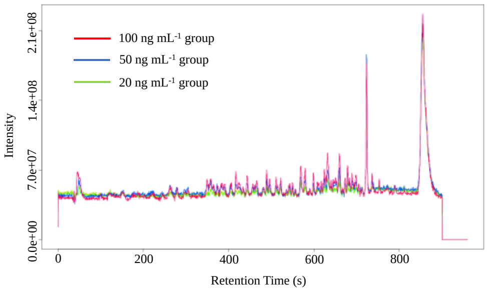

Figure S1. Total ion chromatograms (0 ~ 900 s) of spiked maize sample groups on the W4M platform.

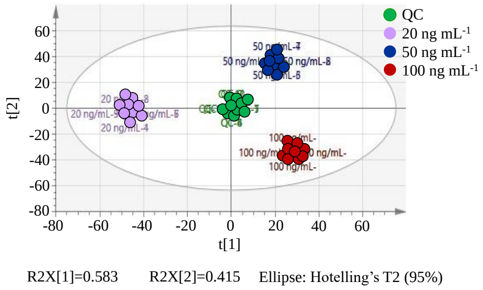

Figure S2. PCA score plot of spiked maize sample groups.

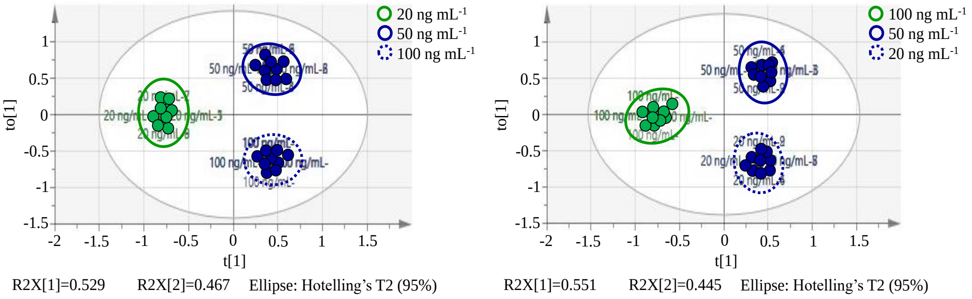

Figure S3. OPLS-DA score plots of spiked maize sample groups.

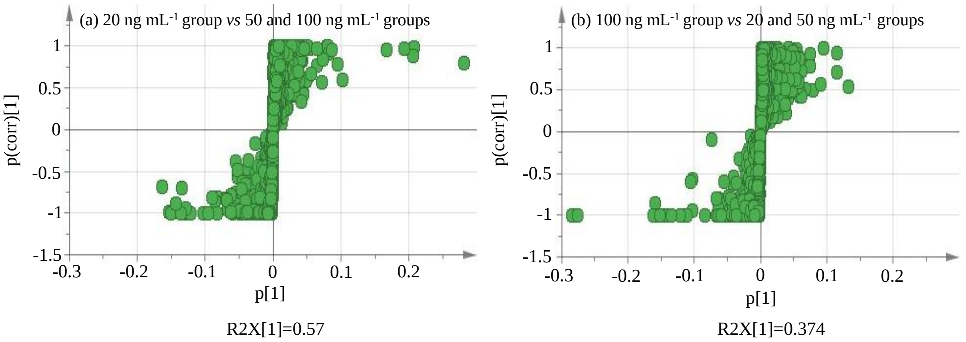

Figure S4. S-plot plots of spiked maize sample groups.

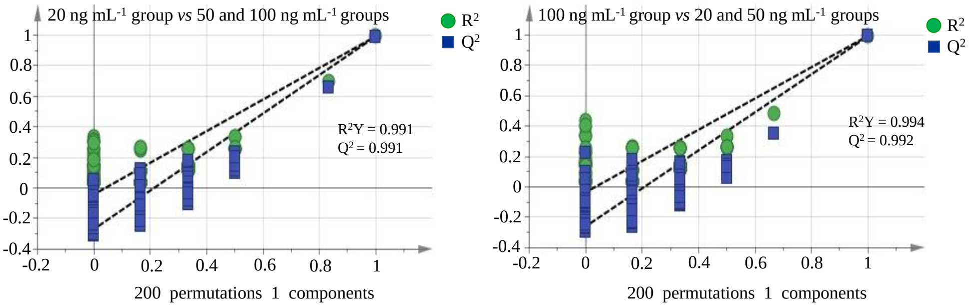

Figure S5. Permutation test plots of spiked maize sample groups.

Table S1. Recovery (%) of spiked ciprofloxacin-d8 in lettuce sample groups (*n*=9).

Concentration groups ($ng\ mL^{-1}$)	1	2	3	4	5	6	7	8	9	RSD (%)
20	84.7	83.5	80.3	80.1	82.1	85.9	83.2	81.3	84.7	2.5
50	81.2	85.4	80.3	80.9	86.2	85.1	86.2	81.4	83.2	2.9
100	87.7	82.1	85.4	87.3	81.6	86.2	87.3	82.1	83.2	3.0

Table S2. Recovery (%) of spiked ciprofloxacin-d8 in maize sample groups (*n*=9).

Concentration groups ($ng\ mL^{-1}$)	1	2	3	4	5	6	7	8	9	RSD (%)
20	88.1	82.7	82.4	87.5	82.3	85.1	86.2	87.5	82.3	2.9
50	83.6	88.9	87.4	81.2	81.3	87.4	88.7	82.3	89.1	4.0
100	81.1	88.2	84.7	83.4	87.6	81.2	82.5	83.4	88.5	3.5

Table S3. Marker compounds screened in maize sample groups.

Var ID (Primary)	Marker compounds	VIP Pred [a]	Coordinate in S-plot [b]	Mass error (ppm) [c]	LOD ($\mu g\ kg^{-1}$)
M214T418	Clorprenaline	3.014/3.007	(-0.077, -0.951)/(0.053, 0.951)	-0.225	0.6
M226T148	Terbutaline	2.021/2.415	(-0.049, -0.954)/(0.045, 0.951)	-1.751	0.3
M228T491	Tolobuterol	3.759/3.263	(-0.096, -0.978)/(0.059, 0.952)	-0.294	0.6
M234T261	Cimbuterol	2.940/4.019	(-0.075, -0.924)/(0.073, 0.968)	-2.061	0.5
M260T571	Propranolol	3.424/4.455	(-0.088, -0.943)/(0.082, 0.957)	0.617	1.6
M273T138	Sotalol	1.237/1.403	(-0.026, -0.955)/(0.018, 0.936)	0.563	1.6
M310T388	Nadolol	3.879/2.097	(-0.099, -0.955)/(0.035, 0.944)	-2.351	0.5
M162T266	5-Chloro-1-methyl-4-nitroimidazole	1.578/1.625	(-0.037, -0.933)/(0.027, 0.929)	-0.394	1.1
M170T467	Ipronidazole	4.251/4.052	(-0.109, -0.946)/(0.077, 0.966)	2.920	0.6
M172T152	Metronidazole	2.552/2.625	(-0.064, -0.952)/(0.047, 0.946)	2.916	0.8
M188T101	Metronidazole-hydroxy	1.902/2.702	(-0.046, -0.915)/(0.049, 0.934)	-0.353	0.6
M201T179	Ronidazole	1.681/1.625	(-0.040, -0.946)/(0.027, 0.923)	-1.134	1.0
M202T351	Thiabendazole	3.593/4.006	(-0.092, -0.917)/(0.069, 0.914)	-1.654	0.7
M220T383	Ornidazole	3.341/1.884	(-0.085, -0.954)/(0.031, 0.938)	-1.476	1.9
M248T298	Tinidazole	2.691/3.536	(-0.067, -0.954)/(0.063, 0.951)	-2.146	0.4
M266T625	Albendazole	3.907/3.189	(-0.100, -0.937)/(0.056, 0.939)	0.143	0.5
M300T661	Fenbendazole	2.764/2.841	(-0.069, -0.966)/(0.050, 0.971)	-0.272	1.0
M262T553	Oxolinic acid	1.815/2.208	(-0.043, -0.948)/(0.037, 0.935)	4.435	0.8
M263T524	Cinoxacin	2.195/2.067	(-0.054, -0.922)/(0.033, 0.954)	2.106	2.1
M320T375	Norfloxacin	2.398/2.065	(-0.059, -0.969)/(0.033, 0.937)	0.600	2.0
M321T381	Enoxacin	2.193/3.428	(-0.055, -0.943)/(0.061, 0.937)	1.102	1.8
M332T406	Ciprofloxacin	2.824/3.537	(-0.072, -0.941)/(0.063, 0.955)	4.829	0.8
M352T427	Lomefloxacin	2.766/2.628	(-0.070, -0.934)/(0.047, 0.954)	1.175	0.4

Table S3. Marker compounds screened in maize sample groups.

M358T420	Danofloxacin	2.556/2.421	(-0.064, -0.938)/(0.045, 0.937)	2.019	2.0
M360T422	Enrofloxacin	2.550/2.347	(-0.064, -0.944)/(0.042, 0.966)	0.770	0.7
M362T386	Ofloxacin	1.277/1.622	(-0.029, -0.931)/(0.026, 0.953)	0.908	0.5
M363T386	Marbofloxacin	1.341/1.626	(-0.031, -0.927)/(0.027, 0.931)	-4.510	0.4
M393T500	Sparfloxacin	1.551/2.890	(-0.036, -0.952)/(0.051, 0.956)	-3.455	0.7
M400T446	Difloxacin	1.259/2.263	(-0.029, -0.938)/(0.039, 0.942)	-2.715	1.8
M271T659	Trenbolone	2.791/1.542	(-0.070, -0.941)/(0.024, 0.956)	1.869	0.6
M287T661	Boldenone	1.795/2.346	(-0.043, -0.967)/(0.042, 0.928)	-0.921	0.7
M345T738	Testosterone propionate	2.412/1.628	(-0.060, -0.972)/(0.027, 0.948)	0.122	0.4
M442T663	Deflazacort	1.259/1.495	(-0.027, -0.964)/(0.020, 0.927)	-4.677	0.7
M445T393	Tetracycline	3.761/4.009	(-0.097, -0.919)/(0.070, 0.932)	-0.539	0.6
M461T385	Oxytetracycline	2.422/2.627	(-0.061, -0.956)/(0.047, 0.937)	1.062	1.7
M479T473	Chlorotetracycline	1.904/2.340	(-0.046, -0.921)/(0.041, 0.973)	1.502	2.1
M215T120	Sulphacetamide	1.339/1.418	(-0.031, -0.924)/(0.019, 0.952)	4.277	1.7
M250T278	Sulfapyridine	2.822/4.035	(-0.072, -0.932)/(0.075, 0.938)	-0.686	0.6
M251T200	Sulfadiazine	1.799/1.843	(-0.043, -0.934)/(0.029, 0.941)	0.422	0.4
M256T260	Sulfathiazole	3.594/2.845	(-0.092, -0.962)/(0.050, 0.944)	0.280	0.7
M265T304	Sulfamerazine	2.129/3.429	(-0.053, -0.949)/(0.061, 0.964)	-2.799	1.8
M268T345	Sulfamoxole	3.015/3.262	(-0.077, -0.923)/(0.059, 0.938)	0.002	1.9
M271T366	Sulfamethizole	2.418/1.366	(-0.060, -0.909)/(0.016, 0.944)	-2.372	0.5
M277T482	Sulfabenzamide	1.607/2.365	(-0.038, -0.925)/(0.043, 0.955)	2.561	0.5
M279T222	Sulfmethazine	3.833/3.069	(-0.098, -0.943)/(0.054, 0.956)	-4.681	0.7
M279T374	Sulfisomidine	2.425/3.607	(-0.060, -0.937)/(0.066, 0.933)	-3.077	1.7
M285T407	Sulfachloropyridazine	1.363/1.519	(-0.032, -0.926)/(0.022, 0.942)	-2.350	1.6
M291T360	Trimethoprim	1.676/1.537	(-0.040, -0.918)/(0.023, 0.937)	0.465	0.9
M301T557	Sulfaquinoxaline	1.553/2.065	(-0.037, -0.956)/(0.033, 0.926)	-0.561	1.1
M336T672	Sulfanitran	1.259/1.518	(-0.028, -0.923)/(0.022, 0.943)	2.996	0.6

Note: [a] two VIP values from 100 and 20 ng mL^{-1} groups, respectively; [b] two-group coordinate values from 100 and 20 ng mL^{-1} groups, respectively; [c] Mass error (ppm) = (extracted molecular weight from W4M platform - extracted molecular weight from LC-MS/MS)*10^6/extracted molecular weight from LC-MS/MS.
